# Optimization of Wide-Field ODMR Measurements Using Fluorescent Nanodiamonds to Improve Temperature Determination Accuracy

**DOI:** 10.3390/nano10112282

**Published:** 2020-11-18

**Authors:** Tamami Yanagi, Kiichi Kaminaga, Wataru Kada, Osamu Hanaizumi, Ryuji Igarashi

**Affiliations:** 1 Division of Electronics and Informatics, Graduate School of Science and Technology, Gunma University, Kiryu, Gunma 376-8515, Japan; yanagi.tamami@qst.go.jp (T.Y.); kada.wataru@gunma-u.ac.jp (W.K.); 2 Institute for Quantum Life Science, National Institutes for Quantum and Radiological Science and Technology, Inage-ku, Chiba 263-8555, Japan; kaminaga.kiichi@qst.go.jp; 3 National Institute for Radiological Sciences, National Institute for Quantum and Radiological Science and Technology, Anagawa 4-9-1, Inage-ku, Chiba 263-8555, Japan; 4 JST, PRESTO, 4-1-8 Honcho, Kawaguchi, Saitama 332-0012, Japan

**Keywords:** fluorescent nanodiamonds, nitrogen-vacancy centers, nanometer-scale thermometry

## Abstract

Fluorescent nanodiamonds containing nitrogen-vacancy centers have attracted attention as nanoprobes for temperature measurements in microenvironments, potentially enabling the measurement of intracellular temperature distributions and temporal changes. However, to date, the time resolution and accuracy of the temperature determinations using fluorescent nanodiamonds have been insufficient for wide-field fluorescence imaging. Here, we describe a method for highly accurate wide-field temperature imaging using fluorescent nanodiamonds for optically detected magnetic resonance (ODMR) measurements. We performed a Monte Carlo simulation to determine the optimal frequency sweep range for ODMR temperature determination. We then applied this sweep range to fluorescent nanodiamonds. As a result, the temperature determination accuracies were improved by a factor ~1.5. Our result paves the way for the contribution of quantum sensors to cell biology for understanding, for example, differentiation in multicellular systems.

## 1. Introduction

Temperature measurements are essential for determining the physicochemical states of a system. Temperature directly affects the progress of chemical reactions through changes in the rate of chemical reactions or the activation of chemical states. Therefore, we take for granted measurement of temperature in chemical experiments. However, the importance of cell temperature has long been overlooked, even though a cell is a complex body of chemical reactions, because there has been no means for temperature measurement in such microenvironments, for example, a cell organelle. For this reason, until recently, biologists have believed that almost all eucaryotes intracellular chemical reactions proceed at 37 °C. However, since the development of thermosensitive imaging probes based on fluorescent molecules [1,2], mesoscopic (greater than molecular size and less than cell size) temperature distributions have been observed in cells. Accordingly, the effect of mesoscopic cell temperature distributions on intracellular systems now requires consideration [3,4,5].

In recent years, fluorescent nanodiamonds containing nitrogen-vacancy centers (NV centers) have attracted attention as fluorescence imaging probes for the quantitative measurement of mesoscale temperatures [6,7,8,9]. An NV center is a lattice defect consisting of a nitrogen impurity atom adjacent to a vacancy (vacuity of a carbon atom) in a diamond crystal [10]. It has a pair of stable triplet electrons that emit strong and extremely stable near-infrared fluorescence (650–750 nm) after green-light excitation (520–540 nm) [11,12]. Furthermore, the intensity of this fluorescence changes significantly depending on the spin state of the triplet electron, even at room temperature [13]. Therefore, fluorescent nanodiamonds can be used not only as fluorescence imaging probes but also as nanometer-scale versatile sensors via optically detected magnetic resonance (ODMR) measurements [6,14,15,16,17]. However, the use of fluorescent nanodiamond as a tool for studying the effect of intracellular temperature on biological phenomena is still under development because the time resolution and accuracy of the temperature determination are insufficient for wide-field ODMR measurements.

Here, we describe an optimal method to acquire ODMR frequency spectra for temperature measurements using fluorescent nanodiamonds by wide-field fluorescence imaging [7,18]. First, we performed a Monte Carlo simulation to determine the optimal sweep frequency range for the measurement of stable temperature determination by curve fitting with two Lorentzian functions. Then, by applying this optimal sweep range to the acquisition of ODMR frequency spectra by wide-field fluorescence imaging, we demonstrated that temperatures can potentially be determined over a region exceeding 200 × 200 μm with 1K/Hz^1/2^ accuracy.

## 2. Materials and Methods

### 2.1. Preparation of Fluorescent Nanodiamonds

Nanodiamond powder (Micron + MDA 0–0.10 μm; Element Six, Didcot, Oxfordshire, OX11 0QR, UK) was treated by electron irradiation (2 MeV, 1.0 × 10^18^ e^−^/cm^2^), thermally annealed at 800 °C for 2 h under a vacuum, oxidized at 550 °C for 2 h to remove surface graphite, and treated with a mixture of H_2_SO_4_:HNO_3_ (9:1 *v/v*) at 70 °C for 3 days to obtain fluorescent nanodiamonds containing negatively charged NV centers. The fluorescent nanodiamonds obtained were treated with 0.1 M NaOH at 90 °C for 2 h, 0.1 M HCl at 90 °C for 2 h, and then washed with Milli-Q three times. For the experiments, we used the fluorescent nanodiamonds prepared in our previous work [15]. The fluorescent nanodiamonds had a particle size distribution of 50–100 nm and contained on average 40 NV centers per particle [15]. The fluorescent nanodiamonds were placed on a coverslip (#1 thickness; Matsunami) to perform the ODMR measurements.

### 2.2. ODMR Measurement by Wide-Field Fluorescence Imaging

Fluorescence images of the fluorescent nanodiamonds were obtained by an electron-multiplying CCD (EMCCD) camera (Andor iXon DU897; Andor Technology, Belfast, UK) by optical excitation using a 532–nm wavelength green laser (Genesis 532 MX, 1000 mW; Coherent Inc., Santa Clara, CA, USA). Fluorescence from the NV centers was collected using a 20× dry objective (CFI Plan Apochromat Lambda 20x/0.75; Nikon, Tokyo, Japan) at a power density ~1 kW/cm^2^. Excitation light was eliminated by a dichroic mirror centered at 575 nm and a 650 nm long-wave pass filter. During the observations, a microwave frequency sweep was performed. The microwaves were generated by a signal generator (N5172B; Keysight Technologies, Santa Rosa, CA, USA) amplified by a microwave amplifier (ZHL-16W-43-S+; Mini-Circuit, Brooklyn, NY, USA) and applied using a 1.5-turn copper coil with a 1 mm diameter at < 700 mW output power. Each ODMR spectrum was acquired using an integrated intensity of all the pixels within a 7 μm × 7 μm area. In order for the experiments to evaluate temperature accuracy, we did not use a stage-top temperature control to avoid periodic temperature fluctuation caused by feedback regulation. Instead of the local temperature control, we controlled ambient temperature around the experimental setup. Additionally, the setup was curtained-off to avoid local air flows. The measurements were performed after 1-h stabilization of temperature. As a result, the temperature changes during 2 min measurements were restricted within ± 0.1 K. The local temperature on the stage top was measured by thermocouple-type thermometer.

## 3. Results and Discussion

**Figure 1:**
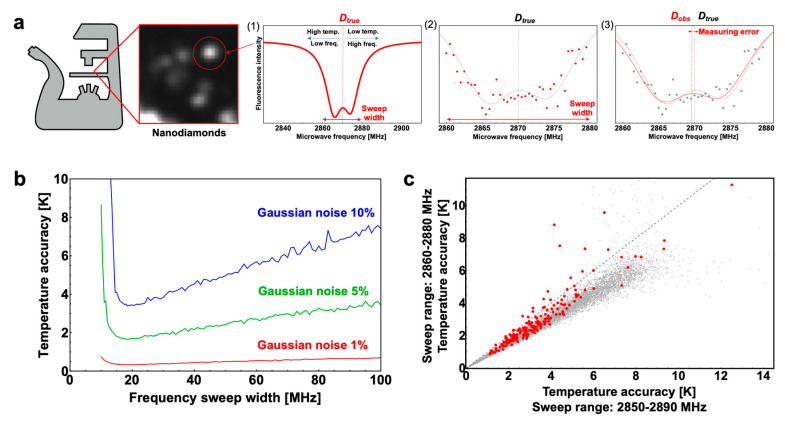
Monte Carlo simulation to determine the optimal sweep range for optically detected magnetic resonance (ODMR) frequency spectrum for temperature determination. (**a**) Schematic outline of temperature determination by ODMR. In the simulation, the ODMR frequency spectrum obtained by a digital frequency sweep during the fluorescence intensity measurements, as shown in (2), was generated at random. (**b**) Simulated temperature measurement accuracy of the frequency sweep width centered at 2870 MHz. The results applied Gaussian noises at 10%, 5%, and 1% of the ODMR signal intensity are shown. (**c**) Comparison of the temperature determination accuracies obtained by the simulation between the sweep ranges of 2850–2890 MHz and 2860–2890 MHz. The red line indicates where the accuracy of the two temperature determinations was equal. Comparison of the temperature determination accuracies obtained through the ODMR measurements between the sweep ranges of 2850–2890 MHz and 2860–2890 MHz were also plotted (red dots).

Fluorescent nanodiamonds can be used as nanometer-scale temperature measurement probes on a fluorescence-microscope stage by applying microwave frequency sweep (Figure 1a). The axial anisotropy parameter *D* of NV centers is approximately 2870 MHz, with an MHz split based on the rhombic anisotropy parameter *E* [13]. The *D* value has remarkable temperature dependence with a proportional constant of 77 kHz/K due to the thermal expansion of the crystal lattice, as shown in Figure 1a (1) [6,19]. To determine the *D* value, an ODMR frequency spectrum was obtained by a digital frequency sweep during the fluorescence intensity measurements (Figure 1a (2)). *D* was then obtained by curve fitting of the observed spectra with a model curve (Figure 1a (3)). However, the determination accuracy of *D* changes measurably depending on the sweep range, even if the spectrum is acquired from the same number of data points, because signal intensity changes occur due to *D* changes only within a limited range of frequency in the ODMR frequency spectrum. To accurately determine temperature using fluorescent nanodiamonds, therefore, the ODMR frequency spectrum has to be acquired using an appropriate sweep range. For this reason, we performed a Monte Carlo experiment to determine the optimal sweep range. We first conjectured that an ODMR frequency spectrum obtained from fluorescent nanodiamonds shows on two Lorentzian functions because an ODMR frequency spectrum without external magnetic fields contains two magnetic resonance signals splitting on the basis of the rhombic anisotropy parameter *E*. Then, we prepared simulated spectra as a model curve according to the following Equation:

(1) $$Fluorescence\;Intensity=1-\frac{a1}{\gamma \pi \left(1+\frac{{\left(-E-D+\omega \right)}^{2}}{\gamma^{2}}\right)}-\frac{a2}{b\pi \left(1+\frac{{\left(E-D+\omega \right)}^{2}}{\gamma^{2}}\right)}$$

where, *ω* is the applied microwave frequency and *a*1, *a*2, and *γ* correspond to the signal amplitudes and the half-width at the half-maximum of the Lorentzian functions, respectively. To provide the parameters for the model curve, ODMR frequency spectra were obtained from 200 fluorescent nanodiamond bright spots. The mean values and standard deviations for each parameter were as follows: *D* = 2869.34 ± 1.31 MHz, *E* = 4.21 ± 0.31 MHz, *a*1 = 0.72 ± 0.16, *a*2 = 0.80 ± 0.23, and *γ* = 5.07 ± 0.59 MHz. Using these parameters for the model curve, we generated 50 points of digital sweep data with various sweep ranges centered at 2870 MHz, added Gaussian noises at random noise levels, and performed curve fitting with the model curve to simulate the observed *D* value (*D_obs_*). We found that at any noise level, the accuracy of the temperature determination was highest using a sweep range of 2860.75–2879.25 MHz (Figure 1b), which was slightly wider than the half-width of the ODMR signal (2*γ* + 2*E* = 18.56 MHz). This means that the accurate temperature could be obtained most accurately using a sweep range that does not entirely cover the signal (2850–2890 MHz), but covers about the half-width of the signal (2860—2880 MHz). The Monte Carlo simulation performed with Gaussian noise at 0–20% of the ODMR signal strength showed that, with a probability of 98.6%, the temperature could be determined more accurately using a sweep range of 2860–2890 MH rather than 2860–2880 MHz (Figure 1c, gray dots).

**Figure 2:**
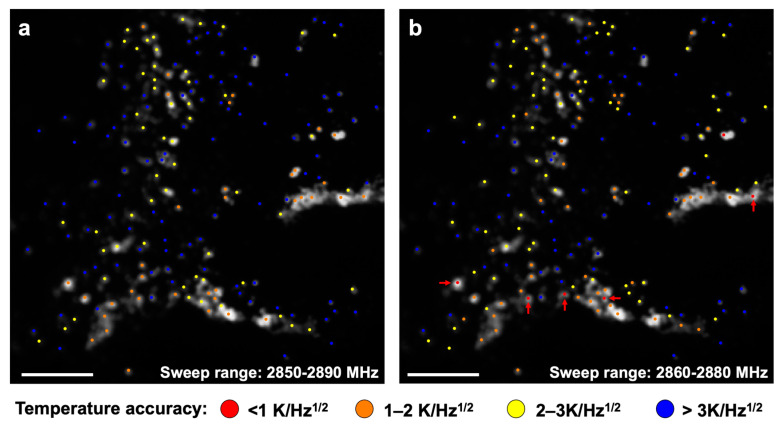
Temperature accuracies obtained by wide-field ODMR measurements of fluorescent nanodiamonds. Fluorescent image of the fluorescing nanodiamonds. The ODMR frequency spectra were obtained at the bright spots indicated by the colored dots. Temperature determination accuracies at each of the bright spots from the (**a**) 2850–2890 MHz and (**b**) 2860–2880 MHz sweeps are shown. Scale bar: 40 μm.

To demonstrate the results of the simulation, we applied the optimal sweep range to the ODMR measurement of the fluorescent nanodiamonds. Fluorescent nanodiamonds were placed on a cover glass, as shown in Figure 2a. The ODMR frequency spectra were then simultaneously obtained from 195 bright spots (colored dots in Figure 2a,b) in a 210 × 210 μm region by wide-field fluorescence imaging. We obtained the ODMR spectra with a sweep range of 2850–2890 MHz using a 100-point digital sweep (sweep time = 2 s) to compose two 50-point (=1 s) spectra with sweep ranges of 2860–2880 MHz and 2850–2890 MHz. By fitting the two spectra with the curve Equation (1), we obtained two *D_obs_* values corresponding to the two sweep ranges at each bright spot. The *D_obs_* values were determined 100 consecutive times to calculate the standard deviations to evaluate the accuracy, from which the temperature determination stability was ascertained by 1 s measurements. A tendency similar to that of the simulation was observed, and 83.7% of the bright spots showed greater temperature determination accuracy using a sweep range of 2860–2880 MHz compared to the 2850–2890 MHz sweep range (Figure 1c). Temperature determination accuracies of 3 K/Hz^1/2^ and 2 K/Hz^1/2^ or higher were obtained for 120 and 59 bright spots, respectively, by the 2860–2880 MHz sweeps; whereas, such high accuracies were obtained at 92 and 36 bright spots by the 2850–2880 MHz sweep (Figure 2a,b). In addition, six bright spots showed a temperature determination accuracy of 1 K/Hz^1/2^ or higher for the 2860–2880 MHz sweep; however, no bright spots showed such a high accuracy for the 2850–2890 MHz sweep.

**Figure 3:**
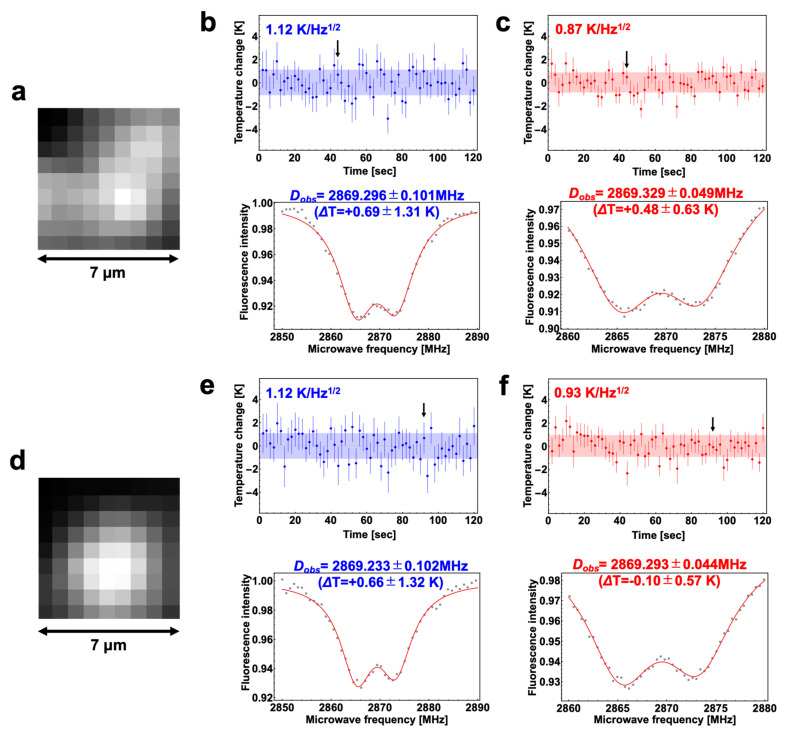
Two typical results of the temperature determination accuracy evaluation at the bright spots A and B are shown in Figure 2a. Fluorescence images (**a** and **d**), the time courses of the temperature changes (top panels in **b**,**c** and **e**,**f**), and typical ODMR spectra (bottom panels in **b**,**c** and **e**,**f**) of A and B are shown. The sweep time of each of spectra was 1 s. The typical spectrum was obtained at the time indicated by the black arrows in the corresponding top panels. The accuracies evaluated by fitting errors are shown in the upper left of the top panels. *Δ*T is the temperature change and the determination error at a measurement point of time, which were evaluated based on *D_obs_* and the fitting error in the determination of *D_obs_*, respectively.

Figure 3 shows two typical results that showed a temperature determination accuracy of 1 K/Hz^1/2^ or higher. Figure 3a–c and Figure 3d–f correspond to the A and B bright spots shown in Figure 2a, respectively. At bright spot A (Figure 3a), the temperature determination accuracy was 1.12 K/Hz^1/2^ using the 2850–2890 MHz sweep range (Figure 3b top). Predictably, the accuracy was improved to 0.87 K/Hz^1/2^ by the 2860–2880 MHz sweep (Figure 3c top). At bright spot B (Figure 3d), the temperature determination accuracy was 1.12 K/Hz^1/2^ using the 2850–2890 MHz sweep range (Figure 3e top), whereas the accuracy was improved to 0.92 K/Hz^1/2^ using the 2860–2880 MHz sweep range (Figure 3f top).

The temperature determination accuracy of each spectrum was also determined by obtaining the standard error of the curve fitting (see the error bars in the bottom panels in Figure 3b,c,e,f). The values at each of the two bright spots were as high as 0.63 K/Hz^1/2^ and 0.57 K/Hz^1/2^, respectively, by the 2860–2880 MHz sweep (Figure 3c,f bottom panels at the time indicated by the black arrows). The accuracies of the same times were 1.31 K/Hz^1/2^ and 1.32 K/Hz^1/2^, respectively, by the 2850–2890 MHz sweep (Figure 3b,e bottom panels at the time indicated by the black arrows). The temporal averages of the values obtained using the 2860–2880 MHz sweep range were 0.86 ± 0.15 K/Hz^1/2^ and 0.95 ± 0.15 K/Hz^1/2^ at bright spots A and B, respectively; both of which were significantly similar to the accuracy obtained from the standard deviation of the *D_obs_* (Figure 3c,f top panels). Therefore, the temperature determination accuracy obtained by the two procedures was validated.

The results strongly suggested that if measurement noises are sufficiently reduced, temperatures can be determined with 1 k/Hz^1/2^ or higher accuracy over a region of 210 × 210 μm using fluorescent nanodiamonds for wide-field fluorescence imaging. This area can accommodate approximately ten eukaryotic cells. Often, intracellular events cannot be completed within a single cell; they progress by exchanging physical and chemical information between multiple cells. For example, tissues consisting of normal cells maintain homeostasis by interacting with multiple cells via physical and chemical signals, such as cytokines. Disruption of this interaction network owing to canceration and aging is known to break this homeostasis [20]. Until now, the application of fluorescent nanodiamonds to intracellular temperature measurements has been limited to single-cell measurements [7,21]; therefore, wide-field temperature measurements using our method are potentially useful for monitoring tissue homeostasis measuring organelle-level mesoscopic temperatures, such as in single-cell mitochondria. In addition, our method is potentially useful for image cytometry for monitoring the differentiation of a large number of stem cells with high throughput [22].

## 4. Conclusions

We propose a method for highly accurate temperature imaging over a range of 210 × 210 μm by ODMR measurement using fluorescent nanodiamonds. Using a Monte Carlo simulation, we found that 2860–2880 MHz is the optimal sweep range for the ODMR frequency spectrum to determine temperatures by fitting the spectrum with two Lorentzian functions. The result of the simulation was confirmed by experiments using fluorescent nanodiamonds. Moreover, the temperature determination accuracy was found to reach to 1 K/Hz^1/2^. Wide-field nanometer-scale temperature sensing is expected to contribute to advances in cell biology and improvements of image cytometry for monitoring differentiation.
